# Feasibility of Tear Meniscus Height Measurements Obtained with a Smartphone-Attachable Portable Device and Agreement of the Results with Standard Slit Lamp Examination

**DOI:** 10.3390/diagnostics14030316

**Published:** 2024-02-01

**Authors:** Massimiliano Borselli, Mario Damiano Toro, Costanza Rossi, Andrea Taloni, Rohan Khemlani, Shintato Nakayama, Hiroki Nishimura, Eisuke Shimizu, Vincenzo Scorcia, Giuseppe Giannaccare

**Affiliations:** 1 Department of Ophthalmology, University Magna Græcia of Catanzaro, 88100 Catanzaro, Italy; mborselli93@gmail.com (M.B.); costanzarossi9@gmail.com (C.R.); taloni.oculistica@gmail.com (A.T.); vscorcia@unicz.it (V.S.); 2 Department of Ophthalmology, Federico II University Hospital, 80131 Naples, Italy; toro.mario@email.it; 3 Department of Ophthalmology, Medical University of Lublin, 20-093 Lublin, Poland; 4 OUI Inc., Tokyo 160-0022, Japan; rohan@ouiinc.jp (R.K.); p.shintaro@ouiinc.jp (S.N.); hiroki@ouiinc.jp (H.N.); eisuke@ouiinc.jp (E.S.); 5 Yokohama Keiai Eye Clinic, Yokohama 240-0065, Japan; 6 Department of Ophthalmology, Keio University School of Medicine, Tokyo 160-0016, Japan; 7 Eye Clinic, Department of Surgical Sciences, University of Cagliari, 09124 Cagliari, Italy

**Keywords:** tear meniscus height, smart eye camera, slit lamp, fluorescein staining, smartphone

## Abstract

Purpose: We aimed to evaluate the feasibility of using a novel device, the Smart Eye Camera (SEC), for assessing tear meniscus height (TMH) after fluorescein staining and the agreement of the results with measurements obtained using standard slit lamp examination. Methods: TMH was assessed using both SEC and conventional slit lamp examination. The images were analyzed using the software ImageJ 1.53t (National Institutes of Health, Bethesda, MD, USA). A common measurement unit scale was established based on a paper strip, which was used as a calibration marker to convert pixels into metric scale. A color threshold was applied using uniform parameters for brightness, saturation, and hue. The images were then binarized to black and white to enhance the representation of the tear menisci. A 2 mm area around the upper and lower meniscus in the central eye lid zone was selected and magnified 3200 times to facilitate manual measurement. The values obtained using SEC were compared with those obtained with a slit lamp. Results: The upper and lower TMH values measured using the SEC were not statistically different from those obtained with a slit lamp (0.209 ± 0.073 mm vs. 0.235 ± 0.085, *p* = 0.073, and 0.297 ± 0.168 vs. 0.260 ± 0.173, *p* = 0.275, respectively). The results of Bland–Altman analysis demonstrated strong agreement between the two instruments, with a mean bias of −0.016 mm (agreement limits: −0.117 to 0.145 mm) for upper TMH and 0.031 mm (agreement limits: −0.306 to 0.368 mm) for lower TMH. Conclusions: The SEC demonstrated sufficient validity and reliability for assessing TMH in healthy eyes in a clinical setting, demonstrating concordance with the conventional slit lamp examination.

## 1. Introduction

The tear film is a thin layer of fluid that covers the eye’s surface. According to the latest definition of the tear film, it consists of two layers: a muco-aqueous layer and a lipid one [1]. Tear film primarily accumulates at the edge of the lower eyelid, forming an inferior tear meniscus, and during blinking, it is evenly distributed across the ocular surface. Tears drain through the lacrimal punctum, located on the medial side [1]. In a normal context, tear meniscus volume values are higher upon awakening and exhibit a significant decrease as the day progresses. Diurnal variation in tear meniscus volume indicates a consistent decrease in tear volume from morning to evening among the general population [2,3]. Adequate tear production prevents the ocular surface from experiencing desiccation, which can lead to dry eye disease (DED). As highlighted in the definition provided by the International Dry Eye Workshop (DEWS), DED is considered a multifactorial disease of the ocular surface characterized by alterations in the homeostasis of the tear film, featuring ocular symptoms such as itching, burning, and tearing. Tear film instability, hyper-osmolarity, ocular surface inflammation, and neurosensory abnormalities are considered key features in the etiological process [4,5]. The loss of water from the tear film’s muco-aqueous layer and subsequent instability contribute to an increased concentration of tears, one of the key factors of DED. This incremented concentration can initiate an inflammatory response, causing cell damage, especially to the conjunctival cells responsible for mucin production. This reduction in mucin exacerbates the instability of the tear film, perpetuating a detrimental cycle [6,7,8].

One of the main metrics for assessing tear production is the Schirmer test. Differences between the wetted length of sheathed and unsheathed strips and the basal tear production rate have been found [9]. Additionally, the tear break-up time (TBUT) test, in which fluorescein is employed, is widely used to evaluate the stability of the tear film in a subjective manner. With the advent of new imaging techniques, it is now possible to assess this non-invasively, without the need for fluorescein (i.e., via the noninvasive break-up time (NIBUT) test). Moreover, it is possible to analyze other aspects of the tear film, such as the lipid layer, meibomian gland dropout using infrared meibography, bulbar redness, and tear meniscus height (TMH) [2]. It is well established that TMH is a reliable metric in the diagnosis of DED. The value of 0.2 mm is conventionally considered the cut-off, with lower values considered to indicate alterations [10]. The process of calculating TMH can vary significantly depending on the choice of the instruments. In some cases, TMH measurements are manually determined by the examiner, while in other instances, this process may involve an automatic or semi-automatic process [11]. The techniques employed in the literature for this purpose include slit lamp examination, which may or may not involve the use of fluorescein staining, meniscometry, and a tearscope [12,13]. Additional methods include the use of a portable digital meniscometer [14] and anterior segment optical coherence tomography (AS-OCT) [15,16]. Furthermore, the use of a fundus camera equipped with an autofluorescence filter [17] and various all-in-one instruments [18] has been reported. In recent times, there has been a growing interest in leveraging the capabilities of artificial intelligence (AI) and deep learning for automating the evaluation of TMH. These modern methodologies have shown considerable promise in enhancing the accuracy and efficiency of TMH measurements [7,8,9,10,11]. 

Another advancement in this field is the development of the Smart Eye Camera (SEC), a novel portable device designed for ophthalmological examinations. This innovative technology transforms a standard smartphone camera into a tool capable of conducting basic evaluations of the anterior segment, including the ocular surface [19]. The SEC’s portability and ease of use make it a valuable addition to the array of tools available for ocular health assessment, especially in settings where conventional ophthalmic equipment is not readily accessible. It has been demonstrated that the SEC is a reliable instrument for assessing anterior chamber depth, iridocorneal angle, and the density of nuclear cataracts [19]. Furthermore, the results obtained from the evaluation of TBUT and corneal fluorescein staining by means of SEC were considered comparable to those obtained using traditional slit lamp examination. On the contrary, among the main ocular surface parameters, TMH has not been studied and validated [20,21]. The primary benefit of using this portable device is its low cost, justifying its straightforward application in areas with insufficient medical facilities [19,20,22]. 

The goal of the present study is (i) to evaluate the feasibility of using the SEC for TMH assessment with fluorescein staining for healthy patients and (ii) to evaluate the agreement of these results with those obtained using standard slit lamp examination.

## 2. Materials and Methods

This prospective, monocentric study was carried out at the Magna Graecia University of Catanzaro, Italy, over a two-month period between 1 September and 31 October 2023. This research project received approval from our Local Ethical Committee (Comitato Etico Regione Calabria—Sezione Area Centro), ensuring compliance with the ethical standards set forth in the Declaration of Helsinki. Prior to participation, all patients provided written informed consent, agreeing to be included in the study and for data treatment. Patients were recruited from among those attending routine visits at the University’s eye clinic. To be eligible for this study, the subjects had to meet the inclusion criteria, i.e., exhibit no signs of active ocular inflammation; have no history of ocular diseases or surgeries, with the exception of cataract surgery performed at least six months prior to the study; and be between 18 and 60 years old. This specific age range was chosen to exclude the older population, which is more prone to developing senile ocular surface alterations. Additionally, participants were required to have an Ocular Surface Disease Index (OSDI) score below 12, indicating minimal or absent ocular surface symptoms and TBUT > 10 mm to minimize the risk of being predisposed to DED [23,24]. Conversely, the exclusion criteria were as follows: use of ocular hypotensive drops, wearing contact lenses, and a confirmed diagnosis of any chronic ocular disease. This careful selection of participants was designed to ensure a homogeneous study population and reliable results. After fluorescein staining, each patient was subjected to (i) a photographic session using a slit lamp (SL9900 ELITE 5X-D, CSO, Firenze, Italy) with an acquisition system (Phoenix, v.3.6) set at x 8 magnification with a blue cobalt filter and (ii) SEC photography with a blue filter at around 4 mm distance from the corneal apex [17,25]. A 10 mm paper strip stained with sodium fluorescein was placed on the inferior eyelid skin as a calibration marker [7]. The photographic session was performed in a dark room to limit bias due to high illumination [8]. Both upper and lower TMH values were calculated for each eye for all patients. 

### 2.1. Smart Eye Camera

Smart Eye Camera is a portable device that functions as a slit lamp. This device is approved as a medical device in Japan (registration number: 13B2X10198030101), Europe (“CE” mark), Kenya, Vietnam, and Indonesia. The primary structure of the SEC is constructed using a 3D printer and is composed of polyamide 12. This instrument is designed to be attachable to the iPhone 8. (Apple Inc., Cupertino, CA, USA). The video resolution is 1080p at 30 frames per second, equivalent to 2.1 megapixels (2,073,600 pixels) per file. SEC contains customizable lenses such as a convex macro lens (focal length = 20 mm, magnification = ×20) for diffuse illumination, a slit light converter with a fixed width of 1 mm, and a blue filter. The slit light is obtained by a cylinder lens on top of the smartphone camera with an angle fixed at 40°. The blue filter is made of acrylic resin and irradiates blue light at a wavelength of 488 nm [8,17]. The lenses can be manually switched by sliding them along the base structure within the designated guides, ensuring stability during the assessment. This photographic system is designed for user-friendly access via a dedicated application (app). This app enables users to conveniently open their smartphone cameras, facilitating the capture of videos. Additionally, it provides a secure offline/online storage space. Moreover, the app features a consultation section, which is an integral part of its design. This area is tailored for telemedicine, allowing for remote ophthalmological evaluations. Through this platform, users can easily engage in virtual consultations with healthcare professionals, making it a valuable tool for both patients and practitioners in the field of ophthalmology [21,26]. 

### 2.2. Fluorescein Staining Protocol

In the current study, each patient was administered 2 milliliters of a 1% sodium fluorescein solution. This was precisely injected into the lower conjunctival sac utilizing a micropipette, ensuring accurate and consistent dosing for all participants [17]. Following this instillation, a photographic session was scheduled to take place using the SEC. It was timed to occur exactly 3 min after the fluorescein solution’s administration. This timing was crucial to allow the solution to disperse and interact with the eye’s surface appropriately. Subsequently, there was a designated waiting period of 30 min. This interval served as a washout phase, allowing any residual fluorescein to be naturally cleared from the eye. After this period, the fluorescein-staining procedure was repeated under the same conditions as before. Following another interval of 3 min post-instillation, a second photographic session was carried out using the slit lamp [17,27,28]. In all cases, photographs of the eye were taken immediately after a blink [29]. 

### 2.3. Image Analysis

**Figure 1:**
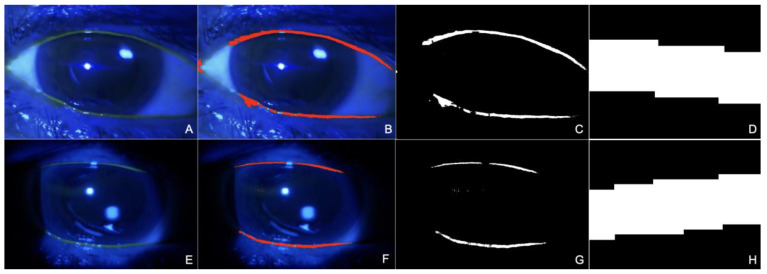
This image presents the results of a detailed analysis of the ocular surface using the two different imaging techniques: the Smart Eye Camera (SEC) and slit lamp examination. The first set of images (**A**–**D**), captured with the SEC, and the second set (**E**–**H**), obtained from the slit lamp examination, each illustrate various stages of the analysis for the same eye. (**A**,**E**) The ocular surface is displayed following fluorescein staining, which highlighted the tear film for better visibility. (**B**,**F**) Focus on the tear meniscus height (TMH), accentuated with a red overlay in color threshold mode to improve visibility. (**C**,**G**) The binarization process involved converting the images into black and white, which enhanced the focus on the tear meniscus. (**D**,**H**) A highly magnified (3200 times) view of a 2 mm area of the upper and lower tear meniscus.

In this study, a comprehensive process was employed for the evaluation of TMH using a suitable protocol for image analysis. All the images were analyzed by a single expert ophthalmologist (M.B.) without masking, as the photos’ origins were easily distinguishable. Each collected image was uploaded to the software ImageJ 1.53t (Image Processing and Analysis in Java, National Institutes of Health, Bethesda, MD, USA). For the analysis of each photograph, a meticulous calibration process was employed. This involved using a 10 mm paper strip, which was stained with sodium fluorescein, as a calibration marker. This marker was fundamental in standardizing the scale of the images by converting the measurements from pixels to a metric scale, thereby ensuring accuracy and uniformity across all images. After setting the scale, a color threshold was applied to the images. This process was conducted using uniform parameters for brightness, saturation, and hue across all photographs. Such uniformity in the application of the color threshold was essential to maintain consistency in the analysis process. Following the application of the color threshold, the images were converted to a binary format, rendering them in black and white. This binarization allowed for the enhancement of the visibility and definition of the tear meniscus in the images, making it easier to identify and measure. For the actual measurement of the tear meniscus, a specific 2 mm area around the upper and lower menisci in the central eyelid zone was selected for examination in each eye. These areas were then magnified 3200 times. Such high magnification was necessary to facilitate precise manual measurement of the tear meniscus height. The measurement process itself involved using a calibrated line, positioned at a 90° angle, to assess the height of the tear meniscus. This method allowed for accurate and reliable measurements, crucial for this study’s objectives. The detailed process and results are illustrated in Figure 1, providing a visual representation of the methodology and findings.

### 2.4. Sample Size

An a priori power analysis was carried out. In this analysis, we utilized the G*Power software product, version 3.1.9.7, a tool for statistical power analysis described in detail by Faul in 2007. The primary objective of this analysis was to accurately estimate the sample size required for the study, ensuring statistical validity and reliability. The power analysis was grounded in data from a study by Niedernolte et al. [15] that involved a total of 20 participants (*N* = 20). This previous research focused on comparing TMH measurements obtained through slit lamp examination and AS-OCT. In the cited study, the effect size was determined to be 0.8, according to the criteria established by Cohen in 1988; this value is considered a substantial effect size in statistical terms. Based on these parameters and by setting a significance criterion (alpha) at α = 0.05 and a power level at 0.80, the power analysis indicated that the minimum sample size required to detect this effect size with adequate statistical power using the Wilcoxon matched-pairs signed rank test was *N* = 15 patients. Therefore, for the current study, a sample size of *N* = 20 patients was deemed more than sufficient to robustly test the study hypothesis.

### 2.5. Statistical Analysis

In this study, the statistical analysis was conducted using GraphPad Prism version 8.2.1 (GraphPad Software, Inc., San Diego, CA, USA). To initiate the analysis, descriptive statistics were systematically computed for all variables involved in the study. For each group within the study, the data underwent analysis using the Kolmogorov–Smirnov test to assess the distribution of the data. The results of the Kolmogorov–Smirnov test confirmed that the data were non-parametric in nature. To compare the values obtained for TMH through the two different methodologies, the Wilcoxon matched-pairs signed rank test was utilized. Furthermore, to evaluate the level of agreement between the two methodologies employed for TMH measurement, Bland–Altman analysis was conducted [30]. This analysis is a statistical method used to compare two different techniques or measurements by determining the agreement between them, thereby providing critical insights into the consistency and reliability of the studied methodologies. For the purposes of this study, the criterion for statistical significance was set at *p* < 0.05.

## 3. Results

**Figure 2:**
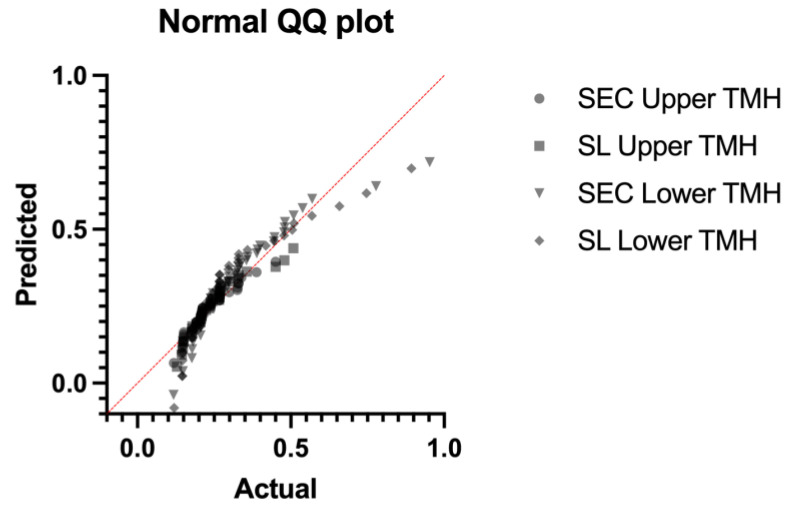
Normality Quantile–Quantile (Q–Q) plot of the data obtained using both instruments for upper and lower TMH. TMH: tear meniscus height; SL: slit lamp.

**Figure 3:**
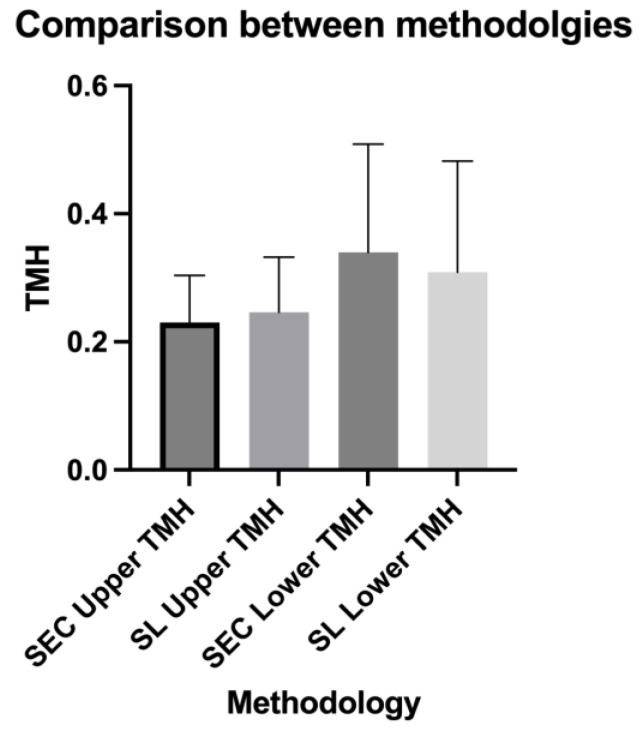
A graphic representation of the results of the Wilcoxon matched-pairs signed rank test, comparing upper and lower tear meniscus height (TMH) values measured using both methodologies and showing no statistical differences for either the upper or lower TMH. The symbol above the columns represents the standard deviation. TMH: tear meniscus height; SL: slit lamp.

**Figure 4:**
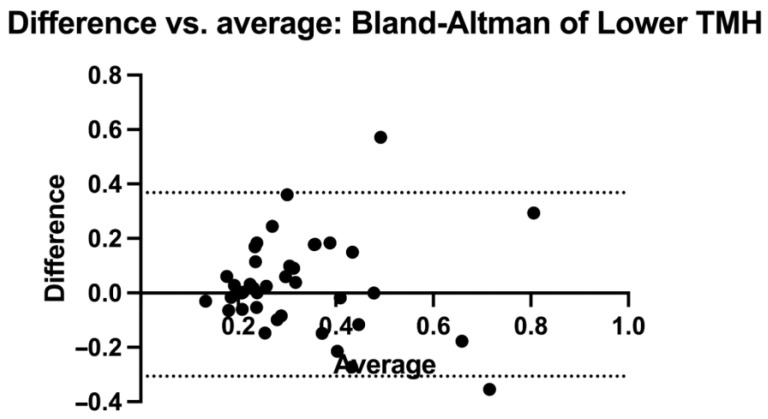
Bland-Altman graphic representation of the lower TMH values for both methodologies. TMH: tear meniscus height.

**Figure 5:**
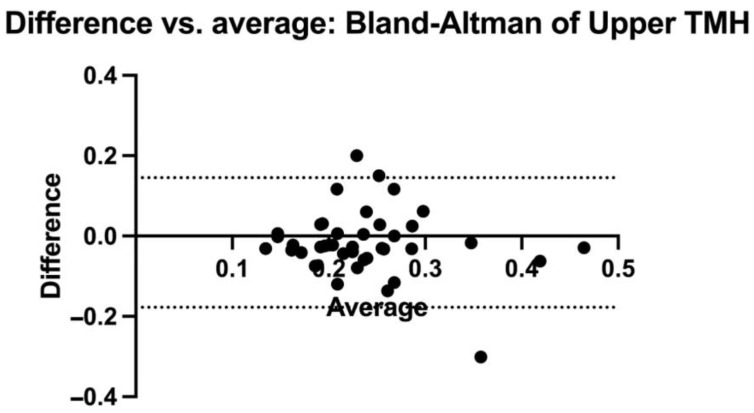
Bland-Altman graphic representation of upper TMH values for both methodologies. TMH: tear meniscus height.

Overall, 40 eyes of 20 subjects (12 males and 8 females; mean age: 52.57 ± 8.16 years) were included in the analysis. The results regarding the descriptive statistics and normality test are presented in Table 1 and Figure 2, respectively. The OSDI and TBUT values were within the normal limits (7.4 ± 3.61; 18.75 ± 3.92, respectively). The upper and lower TMH values measured using the SEC were not statistically different from those obtained using the slit lamp (0.209 ± 0.073 mm vs. 0.235 ± 0.085, *p* = 0.073, and 0.297 ± 0.168 vs. 0.260 ± 0.173, *p* = 0.275, respectively) (Figure 3). The comparison analysis revealed a strong agreement between the SEC and the slit lamp regarding the TMH measurements. For the upper TMH, the mean bias was found to be −0.016 mm, and the 95% limit agreement was found to range from −0.117 mm to 0.145 mm. For the lower TMH, the mean bias was found to be 0.031 mm, and the 95% limit agreement was found to range from −0.306 mm to 0.368 mm (Figure 4 and Figure 5).

## 4. Discussion

This prospective study compared the upper and lower TMH values obtained using a slit lamp, which is considered the current standard for examination, and a novel portable device, the SEC. The results suggest the non-inferiority of the SEC compared to slit lamp examination for TMH evaluation for healthy eyes. The mean bias value indicated a small average difference between the two methods. The limits of agreement were within a statistically acceptable range when considering the small dimensions of the TMH values. In cases of aqueous tear deficiency or Sjögren syndrome, a noticeable reduction in TMH was observed, correlating with a higher incidence of corneal epithelial defects. This reduced TMH is indicative of a diminished tear film layer, which is typically seen in these conditions, leading to increased vulnerability of the corneal epithelium. Conversely, elevated TMH values are often associated with Meibomian Gland Dysfunction (MGD). In MGD, the altered quality of meibomian gland secretions can lead to an unstable tear film, often resulting in an increased tear meniscus [31]. The use of fluorescein solution is a reliable tool for TMH measurement, and a low value obtained using this method is strictly related to DED presence and severity [17]. In the current study, a specific protocol was used to limit the bias due to fluorescein staining in TMH measurement. Moreover, Oguz et al. assessed the lower central lid tear meniscus using meniscometry, both with and without the addition of fluorescein. They concluded that fluorescein had a negligible impact on the reading values and thus is a reliable and easy method for TMH measurement [28]. Regarding the correct timing for acquiring images of these patients, some studies suggested that photographs following fluorescein staining should be captured within 3 to 5 min, but there is no universal consensus on this issue [27,28]. The timing used in the current study was essential to ensure that the images reflected the natural state of the tear film and the eye’s surface immediately following the natural lubrication process induced by blinking. This methodology provided a replicable approach to capturing the effects of fluorescein on the ocular surface, vital for the accurate assessment and comparison of results across all patients assessed in the study. Among all the instruments proposed for TMH evaluation, the lower TMH values were assessed and compared using both a slit lamp and a tearscope. This comparison showed that the tearscope had greater repeatability, and both instruments were found to be reliable [13]. The evaluation of TMH using OCT is considered a highly reliable diagnostic method due to its strong correlation with other established ocular tests and indicators. Specifically, TMH as assessed via OCT shows a significant association with results obtained from the Schirmer test and TBUT. Moreover, TMH measurements correspond closely to subjective symptoms reported by patients, such as dryness, irritation, and discomfort [32]. According to a study by Niedernolte et al., the AS-OCT was a more suitable method for correctly evaluating TMH compared to slit lamp image analysis [15]. Deepening the TMH measurement via OCT, Keech et al. demonstrated that spectral-domain OCT measured significantly higher values than time-domain OCT [29]. Fourier-domain OCT appeared to provide higher TMH values compared to a Keratograph, for which infrared images are used for analysis. Nevertheless, a correlation was found between the TMH level and DED [33]. The same OCT method was used to calculate TMH among healthy children whose values appeared to be lower than those of healthy adults, without a relationship with DED symptoms [26,34]. 

Furthermore, a correlation was found between the use of OCT and a portable digital meniscometer for the measurement of the tear meniscus radius [14]. The aforementioned Keratograph, which is a rapid and non-invasive tool for evaluating various ocular surface parameters, has been identified as a reliable methodology for assessing TMH, demonstrating a strong correlation with established standard methodologies [35]. Currently, AI is extensively utilized in clinical practice to enhance outcomes and reduce bias associated with mechanical measurements, as demonstrated in this context. A study by Wang et al. has shown that the use of an AI-based system is effective in automatically measuring TMH from a provided dataset [36]. It is known that certain conditions can affect TMH evaluation; for example, conjunctival folds can appear at the lower eyelid margin among elderly patients and may lead to an increased TMH measurement. It is necessary for the observer to be aware of this condition and to manually adjust the measurement accordingly [37]

The SEC, an innovative smartphone-attachable device, has been at the forefront of enhancing ocular surface evaluation, enabling measurements of parameters such as anterior chamber depth, angle evaluation, and nuclear cataracts [19,22]. Beyond its diagnostic capabilities, the SEC has also shown excellent interobserver reproducibility in assessing allergic conjunctivitis [38]. Focusing on the parameters of the ocular surface, the SEC has proven to be a reliable instrument for measuring TBUT and corneal fluorescein scores, which are critical in the diagnosis of DED. This has been demonstrated across different models, including murine models and human patients [20,22]. Such versatility is indicative of the SEC’s ability to streamline ocular surface examinations, especially in scenarios where traditional slit lamp microscopy is not available. The SEC represents a viable option for ocular surface workup in the diagnosis of DED, particularly beneficial in remote areas with limited healthcare facilities. Its portability and ease of use also make it an optimal choice for patients who may encounter difficulties in accessing standard eye care services, such as those with reduced mobility or patients who are overweight. Furthermore, the cost-effectiveness of the SEC represents a significant advantage over traditional slit lamp microscopy. This affordability makes the SEC a viable option for widespread use in various healthcare settings, especially in remote outreach programs where resources are scarce [39,40]. Through the SEC, healthcare providers can offer remote consultations, making it a valuable asset in teleophthalmology. The SEC’s design facilitates remote image capture and sharing, enabling ophthalmologists to provide expert evaluations without the need for in-person visits. The collective evidence from these studies underlines the fact that the SEC has the potential to fill the gap in ocular health services by providing an accessible, cost-effective, and reliable alternative to traditional diagnostic equipment. 

Concerning the limitations of this study, it is crucial to highlight the challenge of maintaining the optimal distance between the eye and the lens to capture the most focused image. Additionally, the positioning of the SEC on the right side complicates the evaluation of the left eye due to the obstruction created by the nose; in such instances, rotating the smartphone by 90 degrees to record videos may be beneficial. Another drawback is that TMH values are not instantly available using the SEC, necessitating offline analysis using a personal computer [17,41,42]. For the slit lamp examination, only a blue filter was utilized, and it was not combined with a yellow filter. The concurrent use of both filters has been shown to enhance clarity in the evaluation of corneal details [25]. Nevertheless, the decision to employ solely the blue filter was dictated by the current unavailability of a yellow filter in the SEC. Therefore, our comparison was limited to images obtained exclusively with the blue filter. Furthermore, this study aimed to enroll healthy patients exhibiting normal values of both TBUT and OSDI to evaluate the normal tear meniscus. However, these healthy patients did not undergo additional evaluations for the study of corneal sensitivity and tear osmolarity, among others.

In conclusion, the findings of this study serve to establish that a newly developed instrument, the SEC, is an efficient tool for TMH assessments. Its ease of use and the accuracy of the results it yields make the SEC a valuable asset for TMH assessments in situations where a slit lamp is unavailable. This adaptability and reliability highlight the potential of the SEC as an innovative solution in ocular surface evaluation.

## Figures and Tables

**Table 1:** Representation of descriptive statistics for upper and lower TMH values obtained with both instruments.

	SEC Upper TMH	SL Upper TMH	SEC Lower TMH	SL Lower TMH
Number of values	40	40	40	40
Minimum	0.119	0.129	0.118	0.12
25% Percentile	0.1853	0.1823	0.211	0.2063
Median	0.2095	0.2355	0.297	0.26
75% Percentile	0.2688	0.2698	0.4338	0.3518
Maximum	0.45	0.508	0.953	0.893
Range	*0.331*	0.379	0.835	0.773

TMH: tear meniscus height; SEC: Smart Eye Camera; SL: slit lamp.

## Data Availability

Data are available upon request made to the corresponding author.
